# Caregiver greeting to infants under 6 months already reflects emerging differences in those later diagnosed with autism

**DOI:** 10.1098/rspb.2023.2494

**Published:** 2024-06-14

**Authors:** Aiden Ford, Hasse Walum, Beyonce Brice, Hely Patel, Sanjana Kunnikuru, Warren Jones, Gordon J. Berman, Sarah Shultz

**Affiliations:** ^1^ Neuroscience Graduate Program, Emory University, Atlanta, GA, USA; ^2^ Marcus Autism Center, Atlanta, GA, USA; ^3^ Department of Pediatrics, Emory University School of Medicine, Atlanta, GA, USA; ^4^ Emory College of Arts and Sciences, Emory University, Atlanta, GA, USA; ^5^ Department of Biology, Emory University, Atlanta, GA, USA

**Keywords:** autism, caregiver greeting, development, dyadic interaction, income, infancy

## Abstract

As infants develop, caregivers adjust their behaviour to scaffold their infant’s emerging skills, such that changes in infants’ social abilities are expected to elicit changes in caregiver behaviour. We examined whether changes in the probability of infant-directed caregiving behaviour—specifically, greeting, a ubiquitous signal used by caregivers to initiate reciprocal interactions—differ between infant–caregiver dyads with an infant later diagnosed with autism and dyads with a neurotypically developing infant during infants’ first 6 months. Using longitudinal data from 163 dyads, we found that caregivers in autism dyads (*n* = 40) used greeting less and at later infant ages than caregivers with a neurotypically developing infant (neurotypical dyads, *n* = 83). Caregivers in dyads with infants at elevated familial genetic likelihood for autism who did not receive an autism diagnosis (EL-non-autism dyads, *n* = 40) showed no differences in greeting compared with neurotypical dyads. Socioeconomic status partially mediated the difference between autism and neurotypical dyads. These findings show that autism and socioeconomic status were associated with the mutually adapted dynamics of dyadic interaction beginning in the first postnatal weeks. Importantly, differences in caregiver greeting observed in autism dyads are not interpreted as suboptimal behaviour from caregivers but rather indicate how early emerging social differences related to autism, years before overt features are present, may alter social learning opportunities elicited by the infant.

## 1. Introduction

When interacting with their infants, caregivers adjust their behaviours in response to infants’ emerging skills, optimizing infant engagement and scaffolding new abilities [1–6]. This dynamic adaptation is nicely illustrated by an example from the language domain. Studies of pronoun acquisition show that caregivers initially use their child’s name to function for pronouns when speaking to young toddlers, however in response to their child’s increasingly adept use of self-referential pronouns, caregivers decrease and eventually stop this unconventional use of their child’s name [7,8]. Mutual adaptations like this, which are so fundamental to dyadic interaction, illustrate a key point about developmental change: developmental abilities (e.g. acquisition of pronouns) are neither solely a function of the child nor solely a function of the inputs from their caregiver. Instead, they are best understood as an ongoing, bidirectional process in which a child’s own capacities can influence the experiences provided by their caregiver and social context (and vice versa) [9].

Within the context of neurodevelopmental conditions like autism spectrum disorder (subsequently referred to as autism), it has been proposed that initially subtle differences in infant social ability (e.g. differences in infant social signalling [10,11] or in the infant’s perceived salience of social and non-social information [12–17]) can change the experiential environment elicited by the infant well before diagnostic traits or features of autism are observed [5,18]. These changes would accrue through subsequent development, contributing to further divergences in learning experiences and social ability [18]. In this study, we sought an inroad into this bidirectional developmental process by testing whether caregiver interactive behaviour, measured during infants’ first 6 postnatal months, differs between caregivers of neurotypically developing infants (subsequently referred to as neurotypical) and infants later diagnosed with autism (with neurotypicality and autism diagnoses ascertained at 2–3 years of age). While the vast majority of past work on the early unfolding of autism has focused largely on infant characteristics (reviewed in [19,20]), this study is among the first to examine how infants later diagnosed with autism may elicit different experiences from their caregivers, even in very early infancy. To clarify, we are not suggesting that differences in caregiver behaviour may be contributing to autism. To the contrary, we are suggesting that autism impacts infant–caregiver interactions, leading caregivers to adjust their behaviour (consciously or not) to meet the unique needs and capacities of their infant.

**Figure 1.**
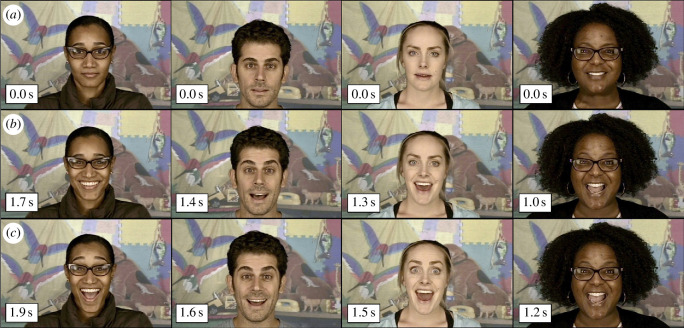
Example still images of greetings from four caregivers. (*a*) Still image from the first frame of the recorded interaction. (*b,c*) Exemplar still images of greeting and the time (from interaction onset) at which it occurred. See electronic supplementary material, video S1 for further examples. All caregivers pictured provided written consent at the time of study enrolment for the use of their image.

Given that differences in dyadic interaction are often reported in autism [21], we focused specifically on changes in caregiver greeting, a ubiquitous caregiving behaviour that plays an important role in initiating dyadic interaction. Caregiver greeting is an infant-directed ostensive cue that engages infant social participation [3,22], increases the salience of subsequently communicated information [23] and enhances learning [24]. A simultaneous widening of the eyes, eyebrows and mouth, caregiver greeting occurs within seconds of seeing their infant and combines mutual gaze with overexaggerated positive affect to instil caregiver’s intention to interact [22,25] (figure 1; electronic supplementary material, video S1). Using a densely sampled, longitudinal dataset of recorded interactions from dyads with neurotypical infants (*n* = 83) and infants on the autism spectrum (*n* = 40), we mapped the probability that caregivers greet their infants from birth to 6 months and tested whether probability of caregiver greeting: (i) increases with infant age (as infants gain increasingly sophisticated social abilities) and (ii) emerges at later infant ages and/or is lower in dyads with an infant later diagnosed with autism compared with neurotypical dyads.

Given the bidirectional, mutually adapted nature of infant–caregiver interactions, changes in the probability of caregiver greeting over infants’ first 6 postnatal months may reflect caregiver perceptions of their infant’s capacity to respond to this highly emotive bid with social signals of their own. For instance, infants who have been consistently responsive to such bids in previous interactions may be more likely to elicit greetings from their caregiver. In neurotypical development, many infant social communicative signals (like smiling and cooing) emerge around 2 months of age [5,26–29] so we hypothesized that the probability of greeting in neurotypical dyads would also increase most rapidly around that age. In contrast, given that autism impacts neurodevelopment as early as the mid- to late foetal period [30], with evidence of differences in social behaviour by as early as 2 months of age [11], we hypothesized that caregiver greeting would emerge at later infant ages and/or that the probability of caregiver greeting would be lower in dyads with an infant later diagnosed with autism compared with neurotypical dyads.

We performed additional control analyses to evaluate whether the hypothesized differences in caregiver greeting between neurotypical and autism dyads could be attributed to between-group differences in caregiver characteristics. Most families in our autism group had an older autistic child (see §2a for details), so their previous experience raising an autistic child may have changed their perceptions of their new infant’s development. Elevated levels of depression and anxiety [31], as well as subclinical autistic traits, are also more commonly reported among parents with an autistic child than in families with only neurotypical children [32–34]. We therefore included dyads with infants at elevated familial genetic likelihood for autism (by virtue of having an older autistic sibling) who did not receive an autism diagnosis (EL-non-autism, *n* = 40) to serve as an important control for many of the potential differences between caregivers in neurotypical and autism dyads. If changes in caregiver greeting are not solely the function of the caregiver, but rather, are influenced by the developing capacities of the infant, then we expected to observe no differences in caregiver greeting between neurotypical and EL-non-autism dyads and significant differences in caregiver greeting between EL-non-autism and autism dyads (with caregiver greeting emerging at later infant ages and/or being reduced in autism compared with EL-non-autism dyads).

Finally, we performed a set of post hoc analyses to assess whether sociodemographic variables reported to influence infant–caregiver interaction [35–37] varied between groups and, if so, the extent to which these sociodemographic differences might account for any observed differences in caregiver greeting between neurotypical and autism dyads. As theorized by ecological models of development [38,39] and documented in the public health literature [40–42], a family’s sociodemographic context influences nearly all facets of their daily experience. Thus, investigating potential associations between sociodemographic factors and caregiver greeting may provide insight into the bidirectional influences of broader structural and societal factors on infant transactions with their familial and social environment [9,43].

## 2. Material and methods

### (a) Participant sample

Study participants were drawn from a larger sample of infants and their primary caregivers who enrolled in the NIH Autism Center of Excellence (ACE) projects at the Marcus Autism Center in Atlanta, Georgia, USA, from 2012 to 2020. Two groups of infants were enrolled: (i) infants with an elevated familial genetic likelihood for autism (EL, *n* = 257), defined as having an older autistic biological sibling and (ii) a comparison group of infants with low familial genetic likelihood for autism (LL, *n* = 297), defined as having a non-autistic older sibling and no autistic family members within three degrees. Autism occurrence for EL infants is approximately tenfold that in the general population [44]. Infants were excluded from ACE project participation for gestational age below 34 weeks, pre- or perinatal complications, major hearing and/or visual impairment, non-febrile seizure disorders or a known genetic syndrome. Families completed demographic questionnaires upon study enrolment, providing information on infant race and ethnicity, maternal education, and household income in the prior year.

Participants were included in the sample for this study if they (i) received a clinical evaluation from 18 to 36 months (described in §2b) and (ii) completed at least one video recording from 0 to 6 months (described in §2d). Dyads were classified into three groups based on infant familial genetic likelihood for autism and diagnostic outcome: (i) LL infants who did not receive a diagnosis of autism or non-autism developmental delay (neurotypical), (ii) EL or LL infants who received an autism diagnosis and (iii) EL infants who did not receive an autism diagnosis (EL-non-autism). The final sample consisted of 83 neurotypical dyads, 40 autism dyads (36 EL and 4 LL) and 40 EL-non-autism dyads (table 1).

**Table 1.** Infant participant sociodemographic information and assessment scores.

	**neurotypical** (*n* = 83)	autism (*n* = 40)	EL-non-autism (*n* = 40)
**infant sex**	40 f, 43 m	13 f, 27 m	14 f, 26 m
**caregiver sex**	82 f, 10 m	40 f, 2 m	37 f, 6 m
**gestational age at birth, mean (s.d.)**	39.0 (1.4)	38.7 (1.6)	39.0 (1.7)
*n*	82	40	36
**ADOS CSS, mean (s.d.)**	1.8 (1.4)^a^ ^b^	6.9 (3.3)^a^ ^c^	2.6 (1.8)^b^ ^c^
*n*	74	40	36
**MSEL, 24 months, mean (s.d.)**			
visual reception AE	29.7 (4.4)^a b^	22.7 (5.5)^c^	26.8 (3.9)^b c^
receptive AE	28.2 (3.5)^a^ ^b^*	17.8 (7.8)^a^ ^c^	26.3 (5.3)^b^ ^c^*
expressive AE	27.3 (6.3)^a^	18.0 (7.4)^a^ ^c^	24.8 (5.8)^c^
fine motor AE	25.7 (3.5)^a^	22.4 (3.4)^a^ ^c^	24.6 (3.1)^c^
gross motor AE	23.1 (3.2)^a^	20.0 (5.1)^a^ ^c^	22.5 (3.0)^c^
*n*	63	38	32
**infant race**	^a b^	^a^	^b^
Asian	0.0%	5.0%	7.5%
Black	3.7%	27.5%	7.5%
White	87.7%	60.0%	72.5%
more than one race	8.6%	7.5%	12.5%
*n*	81	40	40
**infant ethnicity**			
Hispanic or Latine	9.6%	2.6%	7.5%
not Hispanic or Latine	90.4%	97.4%	92.5%
*n*	83	39	40
**maternal education**	^a b^	^a^	^b^
high school	0.0%	7.5%	2.6%
college courses	1.2%	12.5%	2.6%
associate’s degree	2.4%	17.5%	7.7%
college degree	32.9%	40.0%	53.8%
graduate degree	63.4%	22.5%	33.3%
*n*	82	40	39
**household income**	^a^	^a c^	^c^
<$40 000	2.5%	28.6%	2.8%
$40 000–$80 000	15.2%	20.0%	13.9%
$80 001–$100 000	21.5%	14.3%	19.4%
$100 001–$150 000	26.6%	17.1%	44.4%
>$150 001	34.2%	20.0%	19.4%
*n*	79	35	36

^*^ *p* < 0.1

*Notes:* Absence of denotation indicates a non-significant between-group test. *n* denotes the number of infants per group who had the associated assessment or whose families provided the associated demographic information. MSEL scores obtained at 24 months are presented here (as AE scores); standardized *T* scores from the infant’s most recent MSEL (from 2 to 3 years) are available in electronic supplementary material, table S1. Superscripts denote between-group significance (*p* < 0.05) for:

^a^ neurotypical vs. autism;

^b^ neurotypical versus EL-non-autism;

^c^ autism versus EL-non-autism.

ADOS CSS, Autism Diagnostic Observation Schedule, Calibrated Severity 187 Score; MSEL AE, Mullen Scales of Early Learning, Age-equivalent scores.

### (b) Diagnostic procedure

The majority of included infants, 150 of 163, received a clinical evaluation at 2–3 years of age, conducted by expert clinicians who were naive to the infant’s familial genetic likelihood for autism. Assessments included: the Autism Diagnostic Observation Schedule (ADOS; typically ADOS 1 [45], or the Toddler Module [46] or ADOS 2 [47]) to assess social disability, the Mullen Scales of Early Learning (MSEL) [48] to assess developmental level across the Visual Reception, Receptive Language, Expressive Language, Fine Motor and Gross Motor domains, and the Vineland Adaptive Behavior Scales II or III (Vineland II was included in the assessments for the first ACE project and Vineland III was included in the assessments for the second ACE project) [49,50] to assess adaptive function in the child’s daily life . To receive an autism diagnosis, toddlers had to meet the criteria for autism spectrum disorder as defined by the DSM-5 [51]. Neurotypical infants did not have developmental or communication delays as indexed by developmental assessments and evaluated through direct clinical interaction. EL-non-autism infants did not meet diagnostic criteria for autism spectrum disorder as defined by the DSM-5 [51] and determined by clinicians, and received clinical estimates of ‘no clinical features’ or ‘broader autism phenotype’ [52].

Nine neurotypical toddlers and four EL-non-autism toddlers completed their final clinical assessment at 18 months and expert clinicians (also naïve to the infant’s familial genetic likelihood for autism) determined that they did not have autism. Assessments at 18 months included the Communication and Symbolic Behavior Scales (CSBS) [53], Early Screening for Autism and Communication Disorders (ESAC) [54] and the Systematic Observation of Red Flags for Autism (SORF) [55]. Neurotypical infants did not have any reported clinical or parent concerns and did not display developmental or communication delays as indexed by completed assessments and evaluated through direct clinical interaction. EL-non-autism infants did not meet the criteria for autism spectrum disorder as defined by the DSM-5 [51] and few to no concerns were reported by clinicians and parents at evaluation. For this reason, we decided to include these dyads in our sample. Supplemental analyses to assess the extent to which inclusion of infants evaluated at 18 months changed our results are included in electronic supplementary material, figure S2*a,b*; results were unchanged when these infants were excluded.

### (c) Participant diversity statement

White and high-income dyads were overly represented in our sample (table 1, figure 6*a*) (sociodemographic data for Metro Atlanta from the 2020 census in electronic supplementary material, section (*b*), Demographics of Metro Atlanta). Our consent process also required proficiency in English, so our dyads were predominantly English-speaking. We note these differences between our participant sample and the demographics of Metro Atlanta, GA, to acknowledge that systematic barriers to research participation (reviewed in [56]) may have increased sociodemographic homogeneity in our sample.

### (d) Infant–caregiver dyadic interaction

**Figure 2.**
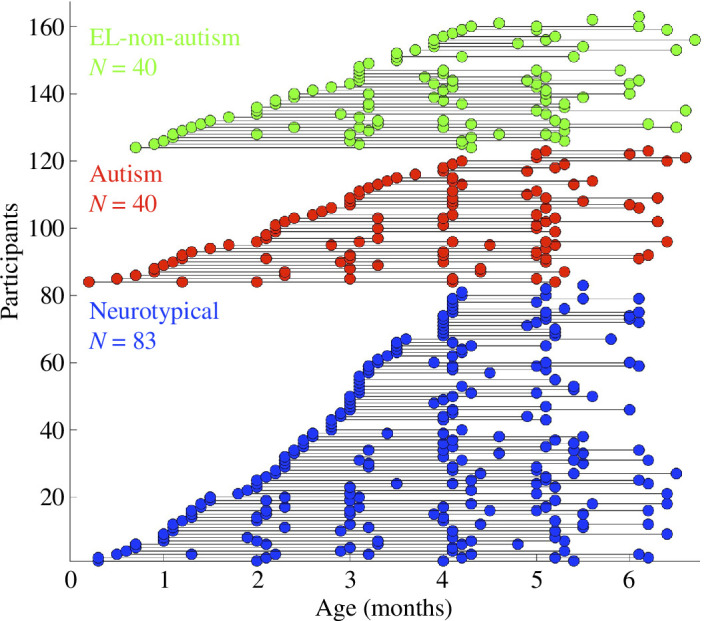
Video recordings of unscripted, screen-mediated interactions were densely and longitudinally collected from infant–caregiver dyads from 0 to 6 months. Distribution of ages at recorded interactions included in analyses for neurotypical, autism and EL-non-autism dyads. Relationship between household income and probability of greeting over infant age.

Recordings of infants and caregivers during a live, screen-mediated interaction were collected from each dyad at up to six timepoints from 0 to 6 months at the Marcus Autism Center (infant age during included sessions visualized in figure 2). Infants and caregivers were seated separately and could see and hear each other in real time. Caregivers were instructed to interact with their infant as they normally would. Details of the recording setup and procedure are included in electronic supplementary material, section (*c*) Description of lab setup for data collection*.* Sessions conducted before 2019 consisted of three sequential 30 s interactions, each recorded separately. Sessions conducted after 2019 consisted of one 70–90 s interaction. In all videos, only the first 4 s were used for analysis. Information on the inclusion criteria for videos and the (non-significant) effect of video order on our analysis is contained in electronic supplementary material section (*d*), Information on the inclusion and exclusion of videos for analysis, and section (*f*), Effect of video number on probability of greeting.

Most infants interacted with the same caregiver at each visit, but a small number (neurotypical *n* = 9, autism *n* = 2, EL-non-autism *n* = 3) were accompanied by an alternative caregiver at some visits. We performed supplemental analyses to confirm that changing caregivers across visits did not influence our results (electronic supplementary material, section (*h*), Effect of diagnostic status on caregiver greeting when visits with an alternate caregiver were removed).

### (e) Behaviour coding

Three trained coders, naive to familial genetic likelihood for autism and diagnostic group, independently recorded the presence or absence of the greeting behaviour from the caregiver in collected videos. Presence of caregiver greeting was identified as a concurrent (i) widening of the eyes, (ii) raising of the eyebrows, and (iii) opening of the mouth more than halfway within the first 4 s of the interaction (see figure 1 and electronic supplementary material, video S1). Sounds made by the caregiver were not included in the criteria to identify greeting. The presence of greeting was coded as a binary variable and coder reliability showed substantial agreement (*κ* = 0.69). Reliability was determined from a random subset of 120 videos (9% of total). This subset of videos was double coded by pairs of raters in which each pair coded a set of 60 videos.

### (f) Statistical approach—sociodemographic information

Chi-square tests were used to assess between-group differences in categorical variables such as infant sex, caregiver sex, infant race, infant ethnicity, maternal education and household income. Kruskal–Wallis tests were used to test for group-level differences in gestational age, ADOS alibrated severity scores and age-equivalent scores across the domains of the MSEL. Post hoc Wilcoxon rank-sum tests were used to isolate between-group differences when appropriate. The results of the described statistical tests are presented in table 1.

### (g) Statistical approach—generalized additive models

To assess how the probability of caregiver greeting changed during infants’ first 6 postnatal months, we constructed binomial generalized additive models (GAMs) in R 4.3.1 (R package *mgcv* 1.8-4.0) [57,58]. In this approach, probability is predicted as a function of infant age and other relevant variables. These predictors can be either continuous and modelled as smooth terms (like age), or categorical and incorporated as fixed terms. Binomial GAMs are generated from fitted splines that are data-driven and highly flexible, which allows for the computation of a functional relationship between predictors and response that is not restricted to standard polynomial or sigmoid shapes. To select the smoothing parameters and optimize model fit, we used the restricted maximum likelihood method.

First, we mapped the probability that caregivers of neurotypical infants would exhibit greeting from 0 to 6 months and assessed the effects of relevant covariates (infant sex, gestational age, caregiver sex) on this trajectory. Second, we investigated whether diagnostic outcome of the infant (neurotypical versus autism) changed the probability that caregivers would use greeting during the first 6 months. We first tested whether there was an overall magnitude difference in the greeting trajectories of the autism and neurotypical groups by modelling diagnostic outcome as a fixed term. Then, to assess differences in the shape of trajectories, essentially an interaction effect between diagnostic outcome and greeting probability over infant age, we created a separate model with diagnostic outcome included as both a fixed and smooth term. In this model, different trajectories were permitted for the neurotypical and autism groups, allowing both trajectory magnitude and shape to vary by group over infant age. Deviance, log-likelihood and model residuals were used to determine how the addition of diagnostic outcome improved model performance. We also analysed the rates of change for each group. We used the same approach to map the trajectories of greeting probability for EL-non-autism dyads.

### (h) Age at first greeting and greeting stability

To test whether greeting emerges at later infant ages in autism dyads, infant age at first caregiver greeting was calculated for each dyad (figure 5*a*). In addition, to examine whether greeting becomes a stable feature of infant–caregiver interactions over infants’ first 6 months, we calculated the percent of interactions in which the caregiver greeted their infant that occurred after the first interaction in which greeting was exhibited (figure 5*b*). Percentages were calculated for individual dyads and ranged from 0% to 100%. For both analyses, we only included infants who came in for their first visit before three months of age (aka prior to the greatest increase in greeting likelihood—see §3a) to avoid inflating group mean values (there were neither between-group differences in age at first visit in the under three-month sample nor in the whole sample; (electronic supplementary figure S1*a,b*)). Final group *n*s for these analyses were neurotypical *n* = 46, autism *n* = 18 and EL-non-autism *n* = 19. We tested for potential group differences using Student’s *t*-tests or Wilcoxon rank-sum tests in MATLAB R2022a (9.12.0.1927505) as dictated by the distribution of the data. Group differences were tested between (i) neurotypical versus autism groups to elaborate on the effect of diagnostic outcome on greeting (figure 5*a*,*b*) and (ii) EL-non-autism versus neurotypical and EL-non-autism versus autism groups to evaluate the effect of possible caregiver characteristics specific to families with a familial genetic likelihood for autism on greeting (figure 5*a*,*b*). Violin plots were made using the Violin Plots for MATLAB GitHub repository from Bastian Bechtold [59].

### (i) Mediation analysis

Finally, to assess whether observed differences in sociodemographic variables measured between autism, neurotypical and EL-non-autism groups (see §2f for details) may be influencing the effects of diagnostic group on greeting observed through the GAM analyses, we conducted a post hoc mediation analysis (R package mediation 4.5.0) [60]. Sociodemographic variables identified for this analysis were those that co-varied with (i) the predictor variable, in this case, a later autism diagnosis and (ii) the response variable, greeting. To co-vary with the predictor variable, the sociodemographic measure needed to differ between the autism and neurotypical groups and between the autism and EL-non-autism groups. These criteria ensured that the mediating variable would represent a sociodemographic context specific to the autism group that would not also track with membership in the broader EL sample (given that 90% of the autism group are EL dyads (see §2a).

The *mediate* function was used to quantify how the addition of sociodemographic variables as covariates influenced the predictive relationship between later autism diagnosis and greeting probability. We evaluated the average direct effect, the average causal mediation effect and the total effect. If the total effect after the mediation analysis was non-significant, later autism diagnosis did not predict greeting probability when sociodemographic variables were incorporated into the model. If the total effect remained significant, the effect of later autism diagnosis on greeting probability could be separated into two sources—the average direct effect, which estimated the predictive effect of later autism diagnosis on greeting probability independent of sociodemographic variables and the average causal mediation effect, which estimated the effect of later autism diagnosis on greeting probability that covaried with sociodemographic variables.

### (j) Data availability

Anonymized data of greeting presence, infant age, gestational age, sex, caregiver sex and sociodemographic variables are reposited at the Open Science Framework [61].

## 3. Results

### (a) Effect of infant age on probability of caregiver greeting in neurotypical dyads

**Figure 3.**
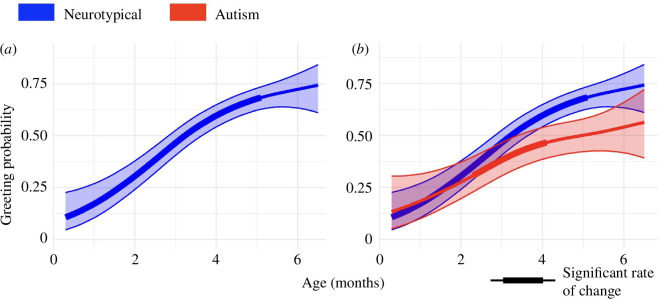
Greeting probability from 0 to 6 months for neurotypical and autism dyads. (*a*) Greeting trajectory for neurotypical dyads. (*b*) Greeting trajectories for neurotypical and autism dyads. In all plots, shading about the mean trajectory shows 95% confidence bands and bolded lines show periods where the rate of change in greeting probability significantly differs from 0.

The probability of caregiver greeting in neurotypical dyads increased from 11% to 74% during the first 6 postnatal months (figure 3*a*). As predicted, the change rate of greeting probability was positive from 0 to 5 months, with a peak increase of 16% per month at 2.8 months. Infant sex (*z* = 1.0, *p* = 0.32), gestational age at birth (*z* = 0.5, *p* = 0.63) and caregiver sex (*z* = 1.4, *p* = 0.16) did not significantly affect the probability that caregivers would use greeting.

### (b) Effect of infant diagnostic outcome (neurotypical versus autism) on probability of caregiver greeting

When later autism diagnosis was included in the GAM, model output, plots and fit statistics showed that diagnosis had a significant impact on the relationship between greeting probability and age. Caregivers in autism dyads used greeting less overall from 0 to 6 months (*z* = 3.0, *p* = 0.003), and this difference was most pronounced in later infancy, from 4.0 to 5.7 months when 95% confidence bands for greeting trajectories of autism and neurotypical dyads were non-overlapping (figure 3*b*). Though the average age of their first visit did not differ between groups (autism 1.6 months and neurotypical 1.6 months, *z* = 0.0, *p* = 0.99) (electronic supplementary material, figure S1*b*), there was a trend towards caregivers in autism dyads first greeting their infants about 3 weeks later than caregivers in neurotypical dyads (3.4 months versus 2.8 months, *t*(62) = 1.4, *p* = 0.09, Cohen’s *d* = 0.4) (figure 5*a*). However, once caregiver greeting was observed in an interaction session, caregivers in both neurotypical and autism dyads continued to greet their infants in interactions occurring at later infant ages (autism 66% and neurotypical 65%, *z* = 0.1, *p* = 0.90). This consistency between autism and neurotypical dyads demonstrated that once initiated, caregiver greeting became a stable feature of dyadic interactions.

### (c) Effect of caregiver characteristics on probability of caregiver greeting

**Figure 4.**
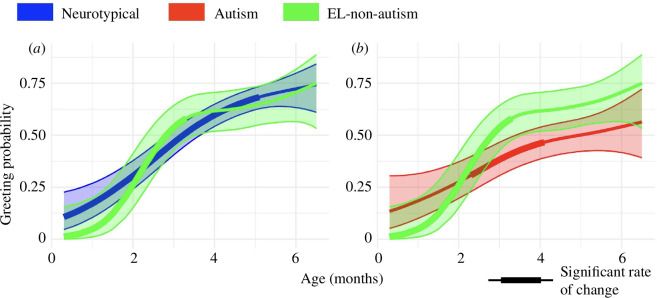
Greeting probability from 0 to 6 months for EL-non-autism dyads compared with neurotypical and autism dyads. (*a*) Greeting trajectory for neurotypical and EL-non-autism dyads. (*b*) Greeting trajectories for autism and EL-non-autism dyads. In all plots, shading about the mean trajectory shows 95% confidence bands and bolded lines show periods where the rate of change in greeting probability significantly differs from 0.

**Figure 5.**
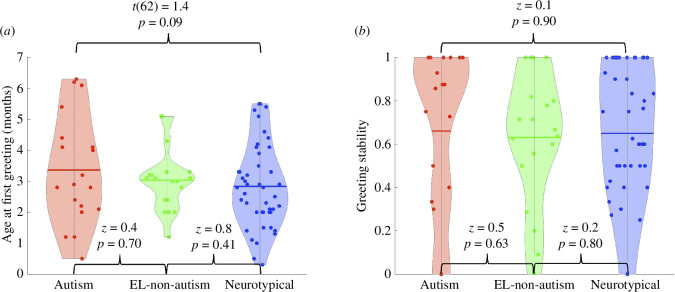
Between-group comparisons of infant age at first greeting and greeting stability. (*a*) Infant age at first greeting. (*b*) Probability that an infant continued to be greeted at subsequent visits after the first visit in which greeting was observed, interpreted as the stability of greeting. The mean value of infant age at first greeting (*a*) and greeting stability (*b*) for each group are marked by the coloured horizontal line and values for individual dyads are marked by the coloured circles. The range and distribution of values from individual dyads are displayed by the violin plot.

As predicted, trajectories of caregiver greeting for EL-non-autism dyads did not differ from trajectories of neurotypical dyads (*z* = 0.4, *p* = 0.71) (figure 4*a*). Caregivers in EL-non-autism dyads also greeted their infants for the first time at an average infant age similar to caregivers in neurotypical dyads (3.0 months and 2.8 months, respectively, *z* = 0.8, *p* = 0.41) (figure 5*a*), and continued to greet their infants after this first greeting with a probability similar to caregivers in neurotypical dyads (neurotypical 65% and EL-non-autism 63%, *z* = 0.2 , *p* = 0.80) (figure 5*b*). These results highlight key similarities in caregiver greeting in the 0–6 month period between neurotypical and EL-non-autism dyads.

In contrast, caregivers in EL-non-autism dyads had a higher probability of greeting from 0 to 6 months compared to caregivers in autism dyads (*z* = 2.0, *p* = 0.049) (figure 4*b*). Statistically significant differences were not present between EL-non-autism and autism dyads in the age at which caregivers first greeted their infants (3.0 and 3.4 months, respectively, *z* = 0.4, *p* = 0.70; figure 5*a*), though mean values showed a decreasing pattern across groups (figure 5*a*). Greeting stability also showed no difference between EL-non-autism and autism dyads (63% and 66%, *z* = 0.5, *p* = 0.63; figure 5*b*). Collectively, these results demonstrate that developmental changes in caregiver greeting were not likely to be associated with the experience of having previously parented an autistic child or other caregiver characteristics specific to families with a familial genetic likelihood for autism.

### (d) Identification of possible mediating variables from between-group differences in sociodemographic measures

**Figure 6.**
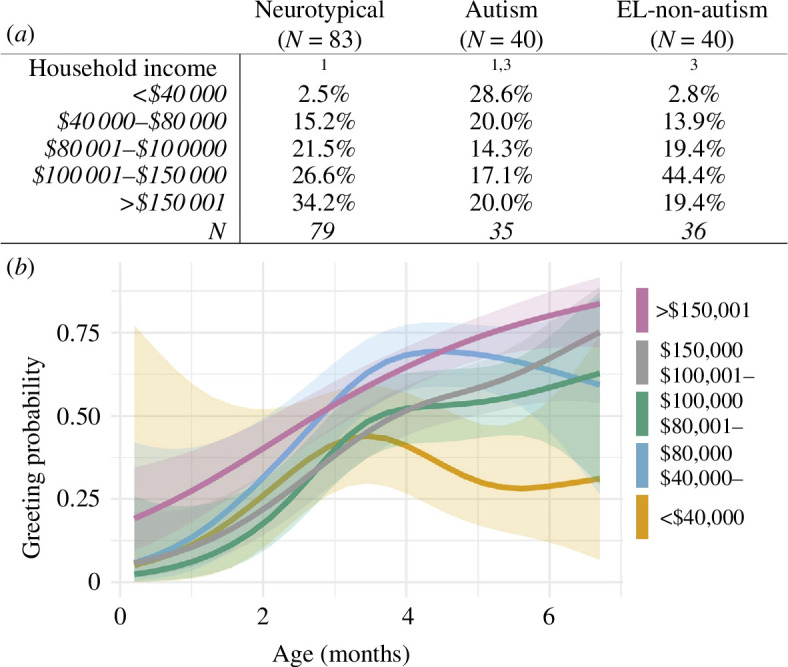
Relationship between household income and probability of greeting over infant age. Neurotypical, autism and EL-non-autism groups were combined and categorized by household income. Coloured lines and shaded 95% confidence bands correspond to greeting trajectories for each income category.

In a post hoc analysis to evaluate whether sociodemographic variables may have mediated the extent to which autism diagnosis predicted decreases in caregiver greeting, we tested for statistically significant differences between neurotypical, autism and EL-non-autism groups in household income in the year prior to study enrolment, maternal education, and infant ethnicity and race. Autism dyads reported significantly lower household incomes during the year prior to study enrolment when compared with both neurotypical and EL-non-autism dyads (autism versus neurotypical *χ* ^2^ = 19.2, *p* = 0.0007, autism versus EL-non-autism *χ* ^2^ = 12.6, *p* = 0.01; table 1 and figure 6*a*). The income distributions of neurotypical and EL-non-autism dyads were not significantly different (*χ* ^2^ = 4.3, *p* = 0.36). Analysis of maternal education and infant race showed that while autism dyads significantly differed from neurotypical dyads (respectively, *χ* ^2^ = 30.1, *p* = 0.000003 and *χ* ^2^ = 19.8, *p* = 0.0002), they did not significantly differ from EL-non-autism dyads (respectively, *χ* ^2^ = 6.7, *p* = 0.16 and *χ* ^2 ^= 5.7, *p* = 0.12). Moreover, EL-non-autism dyads also significantly differed from neurotypical dyads in the distribution of maternal education and infant race (*χ* ^2^ = 11.5, *p* = 0.02 and *χ* ^2^ = 8.0, *p* = 0.046, respectively), reinforcing that between-group differences in these sociodemographic measures tracked with EL group membership rather than later autism diagnosis. To note, all three variables were correlated with each other across the whole sample (electronic supplementary material, figure S3). There were no between-group differences in infant ethnicity (*χ* ^2^ = 1.9, *p* = 0.38). In summary, only household income represented a sociodemographic context that uniquely differed for the autism group versus for membership in the broader EL sample.

### (e) Effect of household income on probability of caregiver greeting

Across all dyads (combining neurotypical, autism and EL-non-autism groups), lower household incomes predicted lower probability of caregiver greeting from 0 to 6 months (*z* = 4.5, *p* = 0.000008; figure 6*b*). Thus, household income in the year prior to study enrolment met criteria to be a potential mediator of the difference in greeting probability between autism and neurotypical dyads: it co-varied with both the predictor variable (later autism diagnosis) and the response variable (greeting probability).

The subsequent mediation analysis assessed the extent to which the difference in greeting between neurotypical and autism dyads was the result of income disparity. Details on the conversion of income from an irregular ordinal variable to an interval variable are described in electronic supplementary material, section (*j*), Details on measurement of household income*.* We found that income accounted for 27% of the difference in greeting between neurotypical and autism dyads. After including income as a covariate, the direct effect of diagnosis (*p* = 0.01) and the total predictive effect (*p* = 0.002) were significant. These findings indicate that later autism diagnosis predicts group differences in greeting trajectories both directly, and indirectly, through household income. Supplemental analyses to further distinguish the effects of autism and household income (see electronic supplementary material, section (*k*), Quantifying the covariate effects of income and diagnostic outcome) confirmed this finding.

## 4. Discussion

Our findings showed that caregiver greeting is associated with emerging differences related to later autism and socioeconomic status, with caregivers of infants later diagnosed with autism and caregivers from lower-income households using greeting less from 0 to 6 months. This finding marks the earliest demonstration of how emerging differences in autism may influence the social interactive environment elicited by the infant, providing a window into the unfolding of autism in early infancy, long before overt features of autism are present.

Inflection points in the trajectory of caregiver greeting aligned with the age at which important social abilities are reported to emerge in infants’ first 6 months. The rate of change in neurotypical caregiver greeting reached a peak of 16% per month in the second postnatal month, coinciding with the age at which infants transform into active and intentional social agents. Infants’ growing ability to reciprocate social exchanges with contingent cues of their own, e.g. caregiver-directed smiling and cooing, results in a qualitative shift in interactions wherein caregivers now recognize their infant as an active social partner [62]. The rate of caregiver greeting slowed after 5 months, coinciding with the period when infants begin to lead interactions [3,63] and can be engaged by more standard, less-exaggerated social cues [22,64,65]. Given that greeting happened within the first seconds of an interaction and once initiated, became a stable feature of subsequent interactions (re-occurring with an average probability of 65%, figure 5*b*), we propose that changes in caregiver greeting index caregivers’ perceptions (conscious or not) of their infant’s readiness to engage as an interactive partner. While immediate cues like infant state or focus of attention may also influence the probability of caregiver greeting, our findings suggest that caregiver greeting is influenced by cumulative experience from past interactions with their infant. Future work may further test this claim by examining how infant signals at interaction onset and infant responses to caregiver greeting influence the probability of caregiver greeting in subsequent interactions.

Caregivers of infants later diagnosed with autism tended to initiate greeting at later infant ages than neurotypical dyads, an average of 3 weeks later, and used greeting less often from 0 to 6 months. This divergent trajectory of greeting is consistent with a recent hypothesis that infant social abilities that emerge at 2 months, especially sensitivity to and preference for social contingencies, may emerge differently in autism [5]. Given the mutually adapted nature of infant–caregiver interaction, differences in infant social engagement specific to autism, altering both infant signals in the moment and cumulative interactive experience, may change how caregivers use infant-directed ostensive cues, like greeting, to scaffold reciprocal interaction.

Importantly, we view these differences in greeting not as a reflection of any suboptimal behaviour on the part of the caregiver, but rather of their ability to adapt their behaviour to the capacities and needs of their infant. Our finding that the greeting trajectory for EL-non-autism dyads did not differ from the trajectory for neurotypical dyads reinforces this interpretation. While a limitation of this study is that we did not collect direct measures of caregiver characteristics, such as depression, anxiety or autistic traits, the similarity between EL-non-autism and neurotypical trajectories demonstrates that caregivers in EL-non-autism dyads who, like caregivers in autism dyads, may have increased familial genetic likelihood for autism and have parented an older autistic child, still adjusted their greeting relative to the experience of interacting with the infant in front of them, a younger sibling who did not later receive an autism diagnosis.

In autism dyads, the decreased probability of greeting suggests that caregivers are seeking other ways of engaging their infant, instead of greeting, that may better reflect their infants’ developmental needs or preferences. This interpretation is consistent with the transactional model of development which proposes that even initially subtle differences in infant social ability may influence the ongoing, bidirectional transactions between an infant’s own capacities and their familial and social contexts—specified here as infant–caregiver dyadic interactions [9,18]. Effected changes to those transactions may affect infant learning experiences, contributing to further divergences in experiential learning and social ability.

Our results therefore suggest that future studies investigating the developmental unfolding of autism might productively focus on transactional processes (i.e. the on-going, bidirectional interactions between the infant and their familial and social context) to illuminate how emerging differences in infant ability alter their experienced social environments, become increasingly divergent trajectories of social development, and ultimately accrue into overt features of autism. Identifying the specific changes to their opportunities for social learning, e.g. how social interactions are cued and initiated, could provide important targets for parent-mediated interventions that seek to support the interaction consequences of early neurodivergences [66]. Importantly, the study of transactional processes is improved by measurement from naturalistic contexts where the dynamic nature of the transaction itself is preserved—here recording, in real time, bidirectional, unscripted interactions between infants and their caregivers.

Finally, lower household income was associated with decreases in caregiver greeting. Autism dyads had lower household incomes than neurotypical and EL-non-autism dyads, which mediated 27% of the difference in greeting between caregivers in neurotypical and autism dyads. As a dimension of the broader sociodemographic context, income influences multiple aspects of the transactional processes unfolding between infants and their familial and social environments [35–37]. Evidence shows that family socioeconomic status, derived from income measures, is associated with variability in structural and functional brain development during infancy (reviewed in [67]), as well as robust differences in trajectories of social communication and cognition [68,69]. Moreover, economic challenges increase the number of stressors placed on a family, as well as the susceptibility to those experienced stressors, which can have downstream effects on mental and physical health and wellbeing [70,71]. Thus, lower income and emerging social disability in autism can both contribute to differences in infant social development which may, as shown by our results, compound changes in caregiver greeting. However, economic status and disability are systemically linked, so their effects cannot be fully disentangled. In the United States, autistic people and their families are more likely to have lower incomes [72–74] and thus disproportionately experience associated economic challenges. Our findings motivate examining how financial options may be included within the broader network of resources aimed at supporting child outcomes [75–77].

In summary, caregivers fine-tuned their greeting, week by week during the first 6 postnatal months, in ways that may match the emerging interactive skills of their infant. Our results reveal that autism and socioeconomic status are associated with how caregivers use greeting, and by extension, the mutually adapted dynamics of dyadic interaction, one of infants’ earliest and most foundational learning experiences.

### (a) Limitations

Our study findings are limited by the demographic and methodological contexts in which we collected our data and by variables not collected for these analyses that we look forward to investigating in future studies. First, as described in §2c, our results overly represent infant–caregiver dyads who are predominantly Western, White, high-income and English-speaking, particularly in our LL sample. To more accurately characterize the independent and combined effects of multiple sociodemographic variables (for instance, race and socioeconomic status (SES)), we advocate that future studies should prioritize the recruitment and retention of samples that are diverse across multiple levels of SES. Second, we analysed singular measures of SES–household income in the past year and maternal education. Future analyses of the influence of SES on dyadic interaction would benefit from more comprehensive measures. Third, information on caregiver autistic traits, anxiety and pre- and ante-natal depression was not collected during this study, so we cannot conclude how caregiver autistic traits or mental health may impact caregiver behaviour or dyadic interactions. Finally, although published studies report few differences between the interactive dynamics of infant–caregiver interactions conducted through screen-mediated versus in-person designs [78–80], we acknowledge that our results are specific to a screen-mediated interactive context. While our experimental setup was constructed to make the screen-mediated interaction as naturalistic as possible (including the use of concealed cameras and microphones to minimize distractions and allow infants and caregivers to see and hear each other in real time, as well as line-of-sight camera positioning to ensure that infants and caregivers were able to look directly into one another’s eyes), future work should examine the dynamics of infant–caregiver interactions in an in-person context where additional cues (e.g. touch, smell, etc.) are present.

### (b) Citation diversity statement

Recent studies have characterized gendered citation biases in many scientific fields [81–83]. To reflect on sources of bias in our own citation practice, we include a field-standard citation diversity statement [84,85] in electronic supplementary material, section (*a*), Citation diversity statement.

## Data Availability

Anonymized data of greeting presence, infant age, gestational age, sex, caregiver sex, and sociodemographic variables are reposited at [61]. Supplementary material is available online at [86].
